# CONGENITAL SYPHILIS IN THE PARAÍBA VALLEY USING A SPATIAL APPROACH

**DOI:** 10.1590/1984-0462/2020/38/2018395

**Published:** 2020-07-29

**Authors:** Luiz Fernando Costa Nascimento

**Affiliations:** a Universidade de Taubaté, Taubaté, SP, Brazil.

**Keywords:** Congenital syphilis, Geographic information system, Social vulnerability, Spatial analysis, Sífilis congênita, Sistema de informações geográficas, Vulnerabilidade social, Análise espacial

## Abstract

**Objective:**

To compare spatial patterns of congenital syphilis (CS) with those of socioeconomic and medical variables in Paraíba Valley, São Paulo, between 2012 and 2016.

**Methods:**

Ecological and exploratory study developed using spatial analysis tools, with information on CS cases obtained from official data reports. Rates were found for CS cases per 1,000 live births, number of family health teams and pediatricians available in the health system per 100,000 inhabitants, and social vulnerability index values. Thematic maps were constructed with these variables and compared using TerraView 4.2.2 software. Estimated global Moran (I_M_) indexes were calculated. In order to detect areas with priority attention regarding the incidence of CS, BoxMaps were developed. The Spearman correlation was estimated for the variable values and compared using the Kruskal-Wallis test. P <0.05 was significant.

**Results:**

144,613 births and 870 CS cases (6.04/1000 live births) occurred during the study period. The average value of CS rates per municipality was 4.0±4.1, (0.0-17.6/1000 live births). Higher CS rates occurred in municipalities of the Upper Vale do Paraíba, contrary to the proportions of pediatricians who were in the far east of the region. The thematic maps of the variables presented a mosaic aspect, which characterized the random distribution of the variables. The I_M_ were not significant. No significant correlation was found between the variables. The BoxMap identified eight municipalities with high CS rates.

**Conclusions:**

Even though it was not possible to identify a spatial pattern of CS rates, it was shown that eight municipalities deserve the attention of city managers.

## RESUMO

**Objetivo:**

Comparar padrões espaciais da sífilis congênita (SC) com os de variáveis socioeconômicas e médicas no Vale do Paraíba paulista nos anos de 2012 a 2016.

**Métodos:**

Estudo ecológico e exploratório, utilizando ferramentas da análise espacial, com informações sobre casos de SC obtidas do Sistema de Informação de Agravos de Notificação (SINAN) e criadas taxas de casos de SC por 1.000 nascidos vivos (NV), de números de Equipes de Saúde da Família e de pediatras atendendo na rede por 100 mil habitantes e de valores do índice de vulnerabilidade social. Foram construídos mapas temáticos com essas taxas, para comparação entre elas, por meio do programa TerraView 4.2.2, e estimaram-se índices de Moran global (I_M_) para essas variáveis. Para detectar áreas com prioridade de atenção para a incidência de SC, foi construído Box Map. Estipulou-se a correlação de Spearman para os valores das variáveis, os quais foram comparados pelo teste de Kruskal-Wallis. Adotou-se erro alfa=5%.

**Resultados:**

Ocorreram 144.613 nascimentos no período e 870 casos de SC (6,04/1.000 NV); o valor médio das taxas por município foi de 4,0±4,1 (0,0-17,6/1.000 NV). Maiores taxas de SC ocorreram em municípios do Alto Vale do Paraíba, ao contrário das maiores proporções de pediatras, que se encontravam no extremo leste da região. Os mapas temáticos das variáveis apresentaram aspecto de mosaico, que caracteriza distribuição aleatória das variáveis, e os I_M_ não foram significativos. Não houve correlação significativa entre as variáveis, e o Box Map identificou oito municípios que apresentavam altas taxas de SC.

**Conclusões:**

Não houve semelhanças entre os padrões espaciais das taxas de SC e demais variáveis, mas identificaram-se oito municípios que merecem atenção dos gestores municipais.

## INTRODUCTION

Between 2012 and 2016, about 98,000 cases of congenital syphilis were reported in Brazil, of which 18,000 were in the state of São Paulo. In 2012, there were 13,000 cases in Brazil and 2,400 cases in the state of São Paulo, and in 2016, there were 25,000 cases in Brazil and 5,000 cases in the state of São Paulo - almost double the number of cases. These values represent rates of 6.1 cases per 1,000 live births (LB).^1^

In 2016 there were 13,000 hospitalizations in Brazil, with costs of R$ 10 million for the Public Health System (*Sistema Único de Saúde* - SUS). There were about 1,900 hospitalizations in São Paulo, which generated about R$ 1.7 million worth of expenses for the SUS.^2^ In the same period, 140 thousand cases of syphilis in pregnant women were reported, of which 7,800 were in the state of São Paulo, almost double the number of cases in Brazil and the state of São Paulo, when comparing the years 2012 and 2016.^3^

Congenital syphilis (CS) is a common sentinel event for monitoring primary health care (PHC), as it is an easily preventable disease, and its occurrence suggests failures in the functioning of the primary health care system and/or its integration with the rest of the health system.^4^ In addition to its effects on mortality, prematurity, low birth weight and acute complications, CS is also responsible for deformities, neurological harm and other sequelae.^5^

The georeferencing of health events is very important for the analysis and assessment of risks to collective health and, through thematic maps, it can explore local and regional determinants of a given event and establish associations between these events and possible associated factors. Given this context, the social vulnerability index (SVI) should be highlighted. It ranges from 0 to 1, and the closer it is to 1, the greater the social vulnerability of a municipality,^6^ which also serves to evaluate interventions.^7^ ^,^ ^8^ ^,^ ^9^

In this regard, the objective of this study was to compare spatial patterns of congenital syphilis rates (per 1,000 LB), SVI values, number of Family Health Teams and pediatricians from each municipality in the metropolitan region of Vale do Paraíba in the years from 2012 to 2016.

## METHOD

An ecological and exploratory study was developed using spatial analysis tools, with information on CS cases in the metropolitan region of the Paraíba Valley and the northern coast of São Paulo, from 2012 to 2016. This region is one of the most industrialized in the state, with an emphasis on the aerospace and automobile sectors. It is made up of 39 municipalities and has a population of over two million inhabitants. Only the municipalities located between the Serra do Mar and the Mantiqueira mountains, which form the Paraíba Valley, were included, excluding four municipalities of the northern coast.^10^ The five-year study period, 2012-2016, allowed for fluctuations in the number of CS cases, either more or less, to be minimized.

The cases identified as CS were obtained from the Reporting Disease Information System (SINAN) regarding the mother’s place of residence.^11^ CS rates were created per 1,000 LB. Information was obtained on the sex of the newborn (NB), and he or she was identified as alive, dead from CS, or dead from another cause. The proportions of pregnant women who attended seven or more prenatal appointments were calculated and information on the number of Family Health Teams in the municipalities of Vale do Paraíba was obtained through the portal of the Department of Informatics of the Public Health System (*Departamento de Informática do Sistema Único de Saúde* - DATASUS), in addition to the number of pediatricians attending in these municipalities.^12^ These last figures were transformed in proportion per 100 thousand inhabitants. Information on SVI was obtained for each municipality included in this study (35 municipalities), and this information was collected from the portal of the Institute of Applied Economic Research (*Instituto de Pesquisa Econômica Aplicada* - IPEA).^6^

The SVI is subdivided into five ranges:

- Very low: Social vulnerability for SVI between 0.000 and 0.200.
- Low: SVI between 0.201 and 0.300.
- Medium: between 0.301 and 0.400.
- High: between 0.401 and 0.500.
- Very high: 0.501 or more.^6^

The spatial analysis consisted of obtaining the global Moran indices (I_M_) for the variables: CS rates, SVI values, proportion of pediatricians per 100 thousand inhabitants and per municipality, and proportion of Family Health Teams (FHT) in each municipality per 100 thousand inhabitants. The I_M_ ranges from -1 to +1, and positive and negative values have positive and negative spatial autocorrelation, respectively.^13^ Values close to 0 indicate no spatial autocorrelation. That is, events are random and, in contrast, values closer to 1 mean greater similarity between neighbors.^13^ The significance level adopted for the analyzes was 5%. The digital grid of the municipalities that make up the Vale do Paraíba micro-regions was obtained from the Brazilian Institute of Geography and Statistics (*Instituto Brasileiro de Geografia e Estatística* - IBGE).^14^

In order to detect areas with higher, lower and intermediate values of the incidence rates of CS, maps were constructed according to the quadrants of the Moran scatterplot (Box Map). In quadrant 1 (high/high), there are municipalities with a high incidence rate and neighbors also with a high rate; In quadrant 2 (low/low), there are municipalities with low incidence rates and neighbors with low rates; in quadrant 3 (high / low), there are municipalities with high rates and neighbors with low rates; and in quadrant 4 (low/high) there are municipalities with low rates and neighbors with high incidence rates. The spatial association in the first two quadrants is positive, with municipalities and their neighbors having similar values. In contrast, in quadrants 3 and 4 the spatial association is negative, with municipalities and their neighbors having different values.^13^

Thematic maps were constructed and compared with the values of the incidence rates of CS, SVI, a proportion of FHT, and a proportion of pediatricians. The incidence rates of CS were analyzed according to the very low, low and medium SVI categories using the Kruskal-Wallis test, and Spearman correlation coefficient values were calculated.

This study was approved by the Research Ethics Committee of the Universidade de Taubaté, under number 009/11.

## RESULTS

In all 35 municipalities of Vale do Paraíba, there were 144,613 births between 2012 and 2016 and 870 cases of CS were identified (6.02/1,000 LB). Of the 870 cases reported, 336 were boys, 481 were girls and 53 did not have this information (6.1% of the reports). According to the SINAN, 782 (89.9%) of these reported cases were alive. In the case of the mothers, 765 had undergone prenatal care, and the diagnosis of syphilis in the pregnant women occurred in 648 cases during prenatal care. The values of the following variables were not considered in this analysis: death from another cause and proportions of pregnant women who had seven or more prenatal consultations. Given the lack of information of these variables, it was not possible to obtain a reliable result.

The average incidence rates, analyzing each municipality individually, for CS and per 1,000 LB was 4.0 ± 4.1, ranging from 0 in nine municipalities (Santo Antônio do Pinhal, São Bento do Sapucaí, Bananal, Arapeí, Areias, Lavrinhas, Queluz, São Luís do Paraitinga and Redenção da Serra) to 17.6 in one municipality (Lagoinha). The average lethality was 11.5±20.3 (ranging between 0 and 100) in 21 municipalities. (Table 1).

The values of I_M_ were not significant (p> 0.05):

- For the CS rate: I_M_=0.05 (p=0.30).
- For FHT rates per 100,000 inhabitants: I_M_=0.02 (p=0.45).
- For pediatrician rates per 100,000 inhabitants: I_M_=0.15 (p=0.08).
- For SVI: I_M_=0.01 (p=0.43).

**Table 1.** Average values with respective standard deviations (SD), minimum and maximum and values of the global Moran index (I_M_), with respective p-value of the congenital syphilis, Family Health Teams, lethality, and pediatrician rates, by municipality of Paraíba Valley, SP, 2012-2016.

	Average (SD)	Minimum-Maximum	I_M_ (p-value)
Congenital syphilis^#^	4.0 (4.1)	0.0-17.6	0.05 (0.31)
Family Health Teams^##^	5.8 (2.7)	1.6-11.8	-0.02 (0.48)
Lethality	11.5 (20.3)	0.0-100.0	-0.03 (0.42)
Pediatricians^##^	20.4 (16.6)	0.0-65.0	0.14 (0.09)

^#^Per 1,000 live births; ^##^per 100 thousand inhabitants.

Such data characterize the absence of spatial autocorrelation. The spatial pattern of these values has a mosaic aspect, and does not identify municipality clusters with similar values (Table 1).

Table 2 shows the consolidated values for all municipalities included in the study for the variables CS, maternal syphilis, number of deaths, number of live NB (according to the diagnosis of CS) and number of Family Health Teams, with average values and respective standard deviations (SD), minimum and maximum values, for each of the 35 municipalities included in the study. The mean values and respective SD of the SVI for the municipalities involved in the research were 0.252 ± 0.050, with a minimum value of 0.177 and a maximum of 0.355; five municipalities presented SVI greater than 0.300, making up the medium vulnerability group: Redenção da Serra, Natividade da Serra, Monteiro Lobato, Cunha and Lagoinha, were all off the Via Dutra axis.

**Table 2.** Congenital syphilis, maternal syphilis, number of deaths, number of live newborns (according to congenital syphilis diagnosis) and number of Family Health Teams, with average values and respective standard deviations (SD), minimum and maximum values, by municipality, Paraíba Valley, SP, 2012-2016.

	Average (SD)	Minimum-Maximum
Congenital syphilis (n=870)^#^	24.9 (65.5)	0.0-330.0
Maternal syphilis (n= 967)	27.6 (64.8)	0.0-342.0
Live NB (n=782)	22.3 (59.1)	0.0-296.0
Deaths (n=88)	2.5 (6.5)	0.0-34.0
Family Health Teams (n=315)	9.0 (12.1)	1.0-55.0

NB: newborns; ^#^all cases.

**Figure 1.**
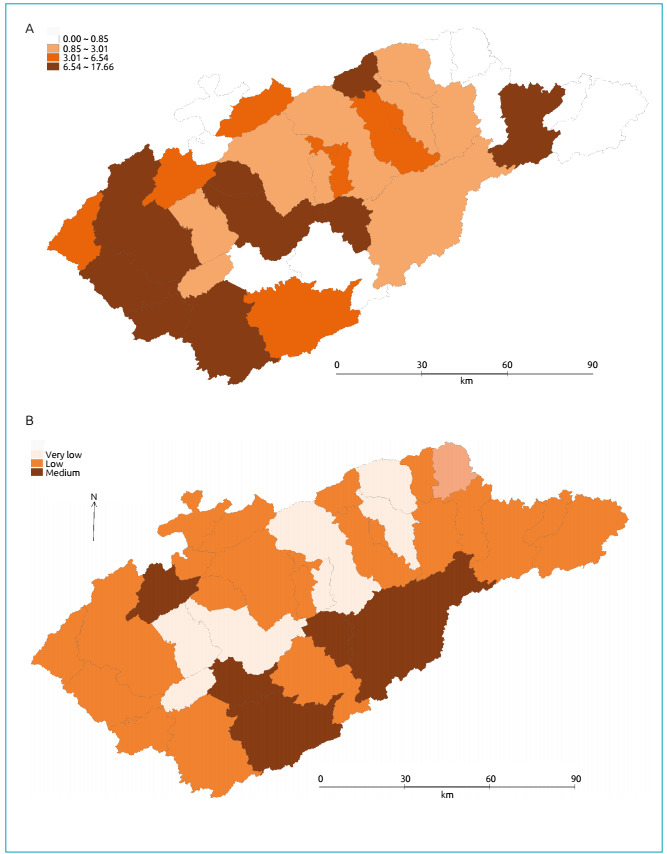
Thematic maps with regard to (A) the distribution of congenital syphilis (per 1000 live births) and (B) the social vulnerability index, Vale do Paraíba, São Paulo, 2012 - 2016.

**Figure 2.**
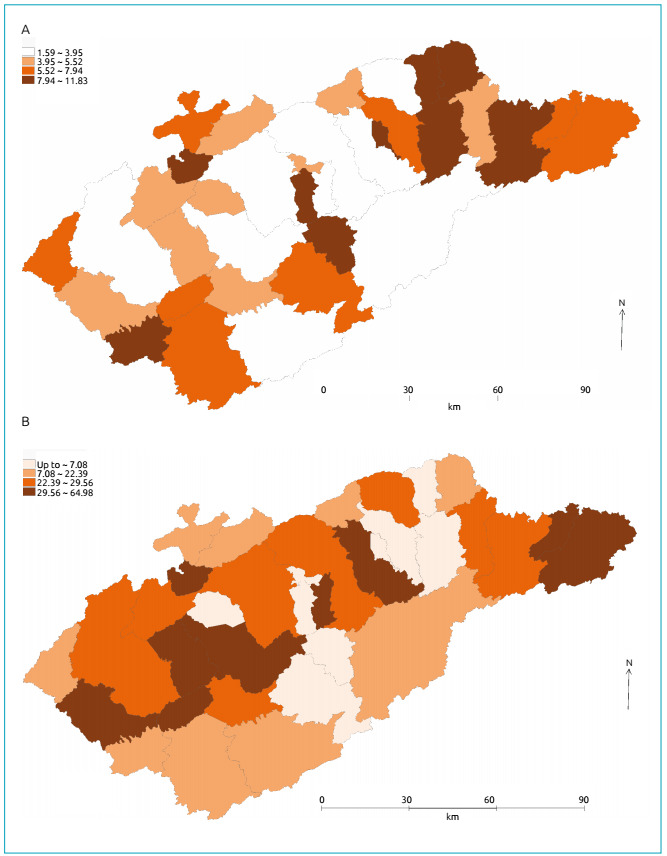
Spatial distribution of (A) Family Health Teams (per 100 thousand inhabitants) and (B) pediatricians by municipality in the Vale do Paraíba (per 100 thousand inhabitants), Vale do Paraíba, São Paulo, 2012 - 2016.

Thematic maps of CS rates, SVI values, pediatrician and family health care ratios are shown in Figures 1 and 2. It can be identified from the thematic map (Figure 1A) that the highest rates of CS are in municipalities of the Upper Valle do Paraíba - São José dos Campos, Jacareí, Santa Branca, Paraibuna, Taubate, Tremembé and Lagoinha - closer to the capital city, all with low SVI, except Lagoinha, which has a medium SVI value. On the other hand, the lowest incidence rates of CS are in municipalities located in the far east of the state and share a border with the state of Rio de Janeiro, except for one municipality, São José do Barreiro, which had a rate in the last quartile (more than seven cases/1,000 LB).

 The thematic map of the SVI values (Figure 1B) for the municipalities of the Paraíba Valley shows very low values in seven municipalities - Jambeiro, Caçapava, Taubaté, Aparecida, Guaratinguetá, Cachoeira Paulista and Cruzeiro -; low in 22 municipalities; and medium in six municipalities - Monteiro Lobato, Redenção da Serra, Natividade da Serra, Lagoinha, Cunha and Queluz -, of which, except for Queluz, are outside the Via Dutra axis. The maximum value was 0.355 for Redenção da Serra, and the lowest was 0.177 for Jambeiro. It was not possible to identify similarities in the CS and SVI maps, as it was expected that municipalities with higher rates of CS cases had worse SVI values.

The spatial distribution of the proportions of Family Health Teams is shown in Figure 2A. The largest proportions are in the east of the metropolitan region of the Paraíba Valley, the so-called Historic Valley, and the smallest are in municipalities along the Via Dutra. Furthermore, there are no similarities between this distribution and the rates of CS.

Figure 2B shows the thematic map of the spatial distribution of pediatrician rates in each municipality. It also has a mosaic aspect, with larger rates in municipalities along the Via Dutra and in some of the far east, and no clustering between them. The values shown by I_M_ already pointed to the absence of spatial autocorrelation for the spatial distributions shown in Figures 1A, 1B, 2A and 2B.

 Spearman’s rank order correlation found no significant values between the incidence rates of CS and SVI values (r=- 0.17), the ratio of FHT programs per 100,000 inhabitants (r=-0.22) and the ratio of pediatricians per 100 thousand inhabitants (r=- 0.05).

When comparing the incidence rates of CS by municipality in three categories of SVI (very low, low, and medium) using the Kruskal-Wallis test, no statistically significant differences were found between them (p = 0.93).

**Figure 3.**
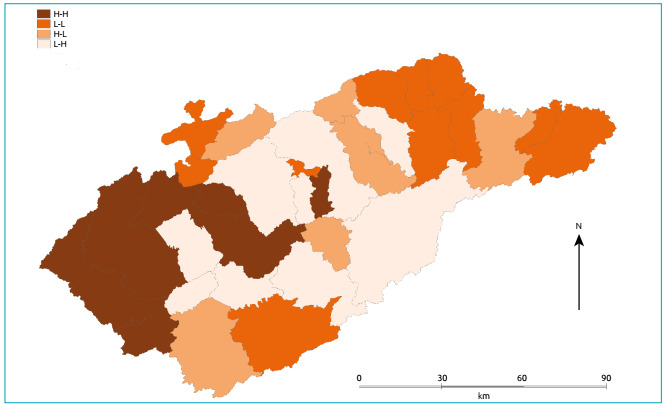
Box Map according to congenital syphilis rates, per 1000 LB, per municipality with the identification of high disease frequency municipalities surrounded by other high disease frequency municipalities (H - H) and low disease frequency municipalities surrounded by other low disease frequency municipalities (L - L), Vale do Paraíba, São Paulo, 2012 - 2016.

The Box Map (Figure 3) identifies eight municipalities (Aparecida, Taubate, Tremembé, Monteiro Lobato, São Jose dos Campos, Igaratá, Jacareí, and Santa Branca) located in quadrant 1 of the Moran (high-high) diagram, which should be investigated for CS rates, as they have high rates, and some are surrounded by municipalities with high rates. The map also identifies 11 municipalities in quadrant 2 (low-low), which are low priority for intervention.

## DISCUSSION

This is the first study on the incidence of CS using a spatial vision in the state of São Paulo. The rate of CS found, according to data obtained from SINAN for the years 2012 to 2016 in municipalities of Paraíba Valley was 6.04/1,000 LB. When analyzed according to each municipality of Vale do Paraíba, these rates range between 0 and 17.6/1,000 LB, with an average of 4.0±4.1 cases per thousand LB in the period. In 2012, the incidence rate of CS in the Paraíba Valley was 2.4/1,000 LB, and in 2016, the rate increased to 10.2/1,000 LB (an increase of ≈330%). In Brazil, the incidence of CS in 2012 was 4.58/1,000 LB, and in 2016 the rate increased to 8.78/1,000 LB, representing an increase of approximately 90%.^15^

A similar approach, a spatial analysis, has been applied in some international syphilis studies.^16^ ^,^ ^17^ ^,^ ^18^ Among the studies conducted in Brazil on this topic, Andrade et al. state that, despite being an old disease and having low-cost treatment, it is still a public health problem. The authors describe a case seen at a university hospital that was not diagnosed during pregnancy because maternal serology was not performed in the last trimester of pregnancy.^19^

A study conducted in Palmas, TO, with data from 2007 to 2014, found 204 cases of CS, with an incidence rate of 2.9/1,000 LB in 2007, and increasing to 8.1/1,000 LB in 2014. The authors raise the possibility of the fragility of health services with regard to the control of CS, as several important flaws were found involving the monitoring of both pregnant women during prenatal and child care, including the inadequate treatment of mothers and their partners.^20^

In Rio Grande do Norte State, research on reported cases of CS from 2007 to 2010 showed that in 2007, the incidence was 2.7/1,000 LB and it increased to 4.3/1,000 LB in 2008 and 2009, dropping to 0.9/1,000 LB in 2010. Despite the fall in the incidence rate, it was higher than 0.5/1,000 LB, which is the target established for the elimination of the disease.^21^

A study with data from the regional health office of Maringá, a city located in the north of the state of Paraná, identified 176 cases of CS, with a progressive annual increase in the incidence of the disease from 0.3 cases per thousand LB in 2011 to 9.7 cases per thousand LB in 2015. The authors point out variables such as age, skin color and low education as associated factors, which were not evaluated in our study due to incomplete information. The authors conclude that there is still a long way to go to eliminate CS.^22^ Other studies describe aspects of case identification, prevalence in pregnant women, treatment, tests performed and prenatal data, as well as characteristics of pregnant women.^23^ ^,^ ^24^ ^,^ ^25^

Our study, in addition to estimating the incidence rate of CS, sought to compare the spatial pattern of these rates with the spatial patterns of SVI distributions and health care indicators (Family Health Teams and number of pediatricians) and, in addition, correlate their values with those variables. There was a negative correlation between SVI values and CS incidence rate, suggesting that the higher the social vulnerability, the lower the rates. However, this correlation was not significant. This is a paradoxical finding, even if this correlation was not statistically significant, since it was expected that the higher the vulnerability and the higher the SVI values, the higher the incidence of CS. Possibly, municipalities with lower SVI and higher rates of CS made more diagnoses because they had better access to health services and better prenatal care, however it was not possible to identify the amount of prenatal consultations, because pertinent information was missing.

Lima et al. identified lack of prenatal care and attention as associated with the occurrence of CS. Such lack of prenatal care may be linked to pregnant women living in locations that are far from basic health units or they are living in poorer regions and, therefore, have more difficulty accessing health services, making them a very vulnerable population.^24^

A study conducted in the city of Rio de Janeiro^9^ using geoprocessing identified 6,274 cases of CS (an incidence rate of 17.3 cases/1,000 LB), with a high proportion of cases whose mothers had low levels of education, were black, and there was a low proportion of pregnant women who attended at least seven prenatal care consultations, suggesting segments of marginalized populations.

In addition to the study by Reis et al.,^9^ in Rio Grande do Sul State, research using spatial analysis tools identified CS rates ranging from 1.0/1,000 LB in 2001 to 5.1 cases per 1,000 LB in 2012, with an annual increase of 0.84 cases per 1,000 LB (p <0.01). The microregions were spatially independent (I_M_= 0.06; p = 0.25), with Porto Alegre having the highest incidence (4.19 cases/1,000 LB) and Jaguarão the lowest (0.23 cases/1,000 LB). Microregions with significant local spatial dependence were observed.^26^

The increase in CS cases highlights a deficit in prenatal care quality. Identifying the micro-regions with the highest incidences is essential in order to bring focus to public policies on this topic. Here, the CS rates were spatially autocorrelated (I_M_= 0.05), but they were not statistically significant (p = 0.31), perhaps because a five-year time series was analyzed and the study cited used 12-year data (2001-2012).

The fact that more populous municipalities located in the Upper Vale do Paraíba have higher rates could be explained by the active search for cases of pregnant women with syphilis, using prenatal laboratory tests with the mother’s treatment. These municipalities have SVI in the very low and low categories. On the other hand, municipalities in the far east, with low and medium SVI values, except São José do Barreiro, have lower rates (first and second quartiles), possibly because they are municipalities with a larger number of FHT staff.

In this article, we sought to correlate CS rates by municipality with the proportions of FHT. Sarraceni and Miranda found a negative, but not significant, correlation between distribution of the observed/estimated ratio of CS and FHT coverage (Pearson’s correlation coefficient, r =-0.40; p=0.51),^27^ which are similar to the findings of this study, conducted in the municipalities of Vale do Paraíba (r=-0.22).

The present study has limitations, highlighting the fact that secondary data were used, which, even from official sources, may contain errors such as incorrect reporting, no reporting, information on maternal age and education, number of prenatal consultations, which may lead to underreporting of the outcome. Even with these possible limitations and the fact that we did not find clusters of municipalities with high or low incidence rates, that is, with spatial dependence between municipalities, it was possible to identify eight municipalities with high incidence rates of CS that deserve attention from local and regional health agencies, in order to identify other variables that may be associated with the outcome studied.
